# Nationwide-free preconception care strategy: Experience from China

**DOI:** 10.3389/fpubh.2022.934983

**Published:** 2022-10-19

**Authors:** Jinghui Xu, Xiaotian Li, Qiongjie Zhou

**Affiliations:** ^1^Department of Obstetrics, Obstetrics and Gynecology Hospital of Fudan University, Shanghai, China; ^2^Shanghai Key Laboratory of Female Reproductive Endocrine-Related Diseases, Shanghai, China; ^3^Institutes of Biochemical Sciences, Fudan University, Shanghai, China

**Keywords:** preconception care, preconception health, reproductive health, China, prenatal care

## Abstract

Preconception care has emerged as a developing field in maternal and child healthcare worldwide. This care type provides couples of reproductive age with the opportunity for early detection and management of biomedical, behavioral, and social health problems. In 2010, the Chinese government launched a nationwide preconception care program as a welfare project. During the past decade, this project has received international attention, and experiences from the project have been published in the literature. In this review, we summarize the history, implementation, and evaluation of preconception care services in China, and its related maternal and children's health service initiatives, to thereby provide knowledge for policymakers and clinicians in other countries.

## Introduction

The preconception period is defined as the 3 months prior to conception (1, 2). It comprises a biological perspective (several days or weeks from gamete maturation to embryo formation), an individual perspective (a conscious intention to have a baby), and a public health perspective (months or years beforehand), in which pre-pregnancy risk factors can be addressed (3). To improve the health of couples with pregnancy intention, increase the chance of having a healthy baby, and optimize pregnancy outcomes, preconception care has been paid more and more attention in recent years. In this review, we summarized the history, implementation, and effectiveness evaluation of preconception care in China, which was initially named as National Free Preconception Health Examination Project, to thereby share the Chinese experience of a nationwide-free preconception care strategy.

It is estimated that in China, more than 54 million fertile couples are older than 35 years, of which ~50% are between 40 and 49 years old (4). With the increasing rate of advanced maternal age and the use of artificial reproductive techniques, the number of reproductive women at risk has increased. It is, therefore, critical to provide couples who are preparing for pregnancy with preconception care, which could help them to avoid potential behavioral and environmental risk factors and achieve the healthiest possible pregnancy (5–8). Preconception care is also an essential part of primary and preventive care (9); therefore, its integration with prenatal care—including prenatal screening and diagnosis as a secondary intervention to form a continuum of care—is expected to further reduce maternal and childhood mortality and morbidity at the population level (10).

Despite its known benefits, the implementation of preconception care varies across the world. In the United States, clinical guidelines related to preconception have recommend that couples with conception intention should have a preconception examination (11). In European countries, such as Belgium, Denmark, Italy, the Netherlands, Sweden, and the United Kingdom, preconception care is offered to women with high-risk factors; however, it tends to be done opportunistically for those without pre-existing medical conditions, except for a national strategy in the Netherlands (12). Maternal and child health have always been a priority in China. Since the launch of the National Free Preconception Health Examination Project in 2010, the Chinese government has been devoted to this nationwide welfare initiative and has achieved some progress, which may set an example for other countries and areas in the world.

## The history of preconception care in China

Premarital medical examinations were used as a prototype of preconception care. During the period 1995–2003, premarital medical examinations were mandatory for couples; however, this requirement was canceled in 2003. Currently, the premarital medical examination is voluntary, and its rate has been reduced by almost 50% (13). In addition, the National Stocktaking Report on Birth Defect Prevention (2012) revealed that the incidence of birth defects was 5.6%, with ~900,000 new cases reported annually in China. This figure has been increasing for 15 years (14, 15). Although neonatal and maternal mortality in China has been continuously decreasing, to 5.4 and 0.169%, respectively, in 2020 (16), the incidence of birth defects in China has plateaued. Thus, the Chinese government initiated free preconception care in the purpose to reduce the incidence of birth defects and other adverse pregnancy outcomes.

The National Free Preconception Health Examination Project was introduced by the State Council of China in 2010 and was operated under the charge of the Chinese National Health and Family Planning Commission and the Ministry of Finance. Well-known experts in the perinatal care field from representative hospitals across the nation brainstormed in a series of meetings to reach a consensus and design this project (17). This well-organized project provides 19 preconception health service items, including health education, health examination, risk evaluation, and medical consultation (17, 18). In particular, the preconception health examinations recommended by expert workgroups are important for obtaining a health profile for further risk evaluation and medical interventions. Specifically, preconception care in China is aimed at couples contemplating pregnancy within the next six months; this is different from the United Kingdom and Australia, where all reproductive couples across the entire reproductive life span are eligible for care (19, 20). In the United States, reproductive planning and health promotion are recommended to be integrated into women's routine healthcare, irrespective of their desire to become pregnant (21).

Initially, the Chinese government provided preconception 164 care as a pilot program in 100 rural counties in 18 provinces, then expanded it to 220 rural counties in 31 provinces after 1 year (17). Free preconception care was also extended to urban communities in some relatively developed regions, such as Guangdong and Chongqing. Hebei, Guangdong, and Gansu provinces have been commended for their service quality, and supervisors from these areas often share experiences with others *via* conference calls (22). Finally, preconception care covered almost all rural and urban areas-−2,790 counties in China–since 2013 (17).

The practical models of preconception care in different areas vary, to some extent. Due to different accessibilities to medical resources, couples living in urban areas are more likely to have preconception care services in tertiary comprehensive or specialized maternity hospitals, while couples living in rural areas usually receive preconception healthcare from trained physicians in primary health centers close to their resident villages and are referred to tertiary hospitals when necessary. Furthermore, additional items related to preconception health examinations are provided where necessary. For example, in Guangdong Province, where incidences of thalassemia and favism are much higher than that in other places, additional screening for these two diseases is routinely performed (23).

While preconception care is a component of regional or state public health programs in most Western countries (9), the Chinese government earmarks a national fund for its free preconception care program as a welfare project to ensure that each couple has the opportunity to achieve a healthier pre-pregnancy condition. The nationwide preconception care service covers more than 80% of couples who plan to conceive within 6 months in China (15), and more than 95% of target couples received preconception health education. From 2010 to 2020, more than 73 million target couples benefited from this welfare project (15–17, 24–27).

## Implementation of preconception care in China

China operates an extensive household registration system, and therefore, basic family information—such as family size and phone numbers within jurisdictions—is available to community workers. These workers can therefore call potential participants, especially married couples with no child, in their local community to determine pregnancy intention and briefly introduce the free national preconception care service. For couples who plan to conceive within 6 months, a health examination would be arranged, with their informed consent (18). In addition, their follow-ups in terms of pregnancy outcomes in future are also arranged *via* telephone.

During the arranged health examination, primary healthcare providers and clinicians who are especially trained in obstetrics collect the following: a thorough individual health history and family history relating to infectious, chronic, or congenital diseases; previous contraception conditions and pregnancy-related difficulties; current medicine use; dietary habits and lifestyles; exposure to toxic substances; and socioeconomic and mental stress (17, 18). A general examination—including height, weight, blood pressure, and heart rate—and a basic physical examination of the body are also performed to determine couples' general health status. Laboratory tests are also a significant component of preconception care in China. A full blood count, blood type identification, urinalysis, liver, kidney, and thyroid function, as well as gynecological ultrasound scans, are routine check-up items in China; these items are quite rare in other country preconception care services (28). Microbiological tests of genital swabs (Candida, Mycoplasma, Chlamydia, and Gonococcus), TORCH (Toxoplasma gondii, cytomegalovirus, and rubella virus), and serum assays for hepatitis B virus and syphilis infection (29, 30) are also performed to determine participants' infection and immunization status. In terms of the examination of sperm, a compact measure to evaluate the husband's reproductivity has been recommended and implemented. Finally, all the records are uploaded to a web-based electronic data collection system.

A novel risk classification system with “ABCDX” categories was generated to stratify couples' preconception health status. In this system, preconception risk factors are categorized into five classes, based on their amenability to prevention and treatment (29). Clinicians then review all health history and diagnostic examination results of the couple to conduct a risk evaluation, based upon which detailed health education, timely patient-centered medical interventions, close supervision during the perinatal period or contraceptive services, and necessary referrals to tertiary hospitals are provided (29, 31).

It is important to build a holistic quality control system for such a large preconception care program. Several guidelines and booklets on health education, health examinations, risk evaluation, and medical consultations have been published (32–37). As 13 of the 19 items provided in this project are laboratory tests, regular training courses for laboratory technicians, as well as internal and external quality assessments of laboratories, were implemented to standardize the operation and to ensure the accuracy of the test results (38). National and provincial quality control centers of laboratories were established for the analysis of the uploaded results from inferior institutions and the arrangement of unannounced inspections.

Recently, a mini mobile application (app) called the “National Premarital and Preconception Health Examination Information Service Platform” was launched to enable easy access for couples planning their pregnancies. This platform contains information on 2,998 institutions that provide preconception care services and automatically recommends the nearest hospital to the participants, based on their location. An appointment can then be made directly (39).

## Evaluation of preconception care in China

Preconception care is believed to have a positive effect on various health outcomes. To evaluate the necessity and effectiveness of preconception care in China, we selected several reports and articles about preconception care based on the Chinese population for this review. Herein, the effect of preconception care on adverse pregnancy outcomes, nutrition intervention, infection and immunization, pre-existing medical conditions, and healthy behaviors is being evaluated.

### Adverse pregnancy outcomes

Studies demonstrate that preconception care services reduce the incidence of maternal and fetal complications. Two intervention studies conducted in Zhejiang Province and the Xinjiang Uyghur Autonomous Region reveal that integrated maternal healthcare (preconception and antenatal care) improved pregnancy outcomes among women of advanced maternal age (≥35 y). The incidence rates of premature delivery, gestational hypertension, gestational diabetes mellitus, low birth weight, and cesarean section were significantly lower among women who received integrated perinatal care (40, 41). This corresponds with the results in other countries, which show reports of 70% lower risk for preterm birth, 60% lower risk of low birth weight and maternal complications, 54% lower risk of neonatal complications, greater odds of obtaining antenatal care, and lower rates of neonatal mortality among women receiving preconception care (8, 42). Meanwhile, the results from the CARNATION study, where a prospective cohort of Chinese women with pre-gestational type 1 diabetes received comprehensive preconception-to-pregnancy management in 11 centers from eight Chinese cities from 2015 to 2017, showed that the continuum of maternal care can achieve substantial improvements in pregnancy outcomes (43). Furthermore, a recent Chinese birth cohort study reports that the prevalence of birth defects is 2.5% (44), which is a significant decrease from 5.6% (14). The improvement shown in this prospective longitudinal mega cohort may be related to the nationwide-free preconception care service system, which could provide timely primary intervention, to some extent. However, further cohort studies are required to assess the contribution of preconception care in improving pregnancy outcomes.

### Nutrition intervention

Nutrition is one of the most common modifiable factors related to conception (45). In the Chinese preconception care project, weight control, folic acid intake, and anemia were the three key factors.

First, abnormal preconception maternal weight is prevalent among Chinese women.

On the one hand, maternal obesity is closely associated with an increased risk of infertility (46, 47), preeclampsia, gestational diabetes (48), and an adverse intergenerational impact on offspring (49). On the other hand, low maternal weight is associated with low birth weight and premature birth (50, 51). In China, the incidence of being overweight and obese among adults has increased to 34.3 and 16.4%, respectively, by 2020 (52). Another study involving 2,120,131 Chinese women—aged 21–49 and from 220 pilot counties in 31 provinces—who participated in the National Free Preconception Health Examination Project from 2010 to 2012 revealed that the prevalence of preconception maternal low BMI increased from 10.4% in 2010 to 14.14% in 2012 (53), especially among women younger than 35 years (51). It is therefore possible that a more complex nutritional challenge, involving malnutrition and overnutrition, is prevalent among Chinese women of reproductive age.

Interestingly, paternal weight may also play a role in offspring birth weight. Guo et al. conducted a retrospective study based on preconception data of 256,718 small for gestational age and 506,495 large for gestational age neonates in more than 4.7 million Chinese couples aged 20–49 between 2013 and 2016. Their results reveal a non-linearly dose–response relationship between lower paternal BMI and small for gestational age, as well as between higher paternal BMI and large for gestational age infants (54). These findings suggest that both ideal maternal and paternal weights seem beneficial for improved pregnancy outcomes and offspring health. It would therefore be meaningful to provide person-centered health education, involving nutritional advice and lifestyle changes, to achieve and maintain a more normal weight before pregnancy.

Second, folic acid supplementation is widely recommended for couples with pregnancy intention to reduce the risk of neural tube defects in their offspring (55). In a cohort who took part in the preconception care program, preconception folic supplementation reduced the risk of spontaneous abortion and preterm birth (56, 57). Moreover, an earlier start of taking folic acid supplements is important. After adjusting for covariates, women with at least three months of folic acid supplementation prior to conception had 10% and 5% lower risks of spontaneous abortion and preterm birth, respectively. Conversely, among women who initiated folic acid supplementation 1–2 months before conception, in 44%, the protective effect of folic acid supplementation against spontaneous abortion still exists after conception, while there was no meaningful reduction in preterm birth. In addition, a review of the association between folic acid and multivitamin supplementation and neurodevelopmental disorders in offspring indicates that supplementation reduced children's risk of autism spectrum disorder by 36% (RR 0.64, 95% CI: 0.46, 0.90) (58). It, therefore, appears to be necessary to strengthen a full course folic acid supplementation during the preconception period to maximize its protective effect.

Third, anemia is common among women of reproductive age and requires early intervention before conception. A routine blood test is performed during the preconception health examination in China to determine maternal iron levels. In a Chinese population-based study in 2012, the prevalence of anemia was 24.80% among women of reproductive age in rural areas (59), while the prevalence of severe anemia was 0.24% (60). Although this incidence was lower than the global average of 29.0%, reported by the World Health Organization in 2011 (61), it varied in terms of different areas and among women of different races in China (62, 63). Between 2014 and 2018, the incidence of anemia in China declined from 23.0% to 16.4% (63), indicating the effectiveness of nutritional strengthening. In addition, there is a U-shaped relationship between hemoglobin concentration and adverse outcomes, according to many studies generated from the National Preconception Health Examination Project in China. Both severe anemia and a hemoglobin concentration higher than 150 g/L prior to pregnancy were associated with an increased risk of spontaneous abortion (64), very early preterm birth (65), low birth weight, and small for gestational age infants (66). Therefore, screening for anemia during the preconception period is of great public health significance to enable timely intervention (67).

### Infection and immunization

Some bacteria, viruses, and other organisms can be passed from mother to child. These are typically TORCH (toxoplasma, rubella virus, cytomegalovirus, herpes simplex virus, and other pathogens) pathogens that can pass across the placenta or the female reproductive tract, thereby greatly increasing the risk of adverse outcomes, such as miscarriage, abortion, stillbirth, sterility, preterm birth, and birth defects—specifically, congenital heart diseases (68, 69). In China, TORCH screening prior to pregnancy makes the preconception period a window to take action to block vertically transmitted infections.

Several articles based on the Chinese population reveal the seroepidemiological status of individuals. The overall prevalence of rubella virus IgG seropositivity increased from 58.4% in 2012 to 81.97% in 2015, and the overall prevalence of rubella virus IgM seropositivity was 0.89% (70, 71). This may be attributed to the nationwide Expanded Programme on Immunization, which includes rubella virus vaccines for adolescents and women of childbearing age in China (72, 73). However, ~20% of women are susceptible to rubella in the preconception period, which requires accelerating the elimination of rubella through supplementary immunization activities to reduce the burden of congenital rubella syndrome (73, 74). The overall IgG seropositivity prevalence of HSV-(1+2) in reproductive-aged women is 90.15% (70). Toxoplasma gondii IgG seropositivity among Chinese women of reproductive age was 1.71–2.3% and that of IgM positive was 0.3–0.4%, which was relatively low (70, 75). Substantial differences among regions in the prevalence of these infectious pathogens are common; this may be related to imbalanced socioeconomic conditions, the availability of health resources, and cultural differences across China. Thus, healthcare service providers should consider local resources and culture when devising preconception care strategies.

### Pre-existing medical conditions

In the preconception period, any comorbidities should be carefully treated and well-controlled. A cross-sectional seroepidemiological survey of preconception hyperglycemia on more than 2.2 million Chinese women revealed that 1.4% and 12.9% of women had diabetes and prediabetes, respectively (76). This indicates the necessity of routine diabetes screening in preconception care. In Guangdong Province, women of childbearing age with type 1 diabetes mellitus had poor awareness of preconception care, with a much lower frequency of self-monitoring of blood glucose than recommended (77), suggesting that preconception glycemic control by appropriate methods is one of the most important aspects of preconception care. In addition to hyperglycemia, abnormal thyroid function is more common during the preconception period. Maternal hypothyroidism is related to preterm birth and impaired neurodevelopmental function (78–82). Sufficient thyroid hormone levels are essential for fetal growth, especially brain development, and timely medication should be initiated and continuously monitored during pregnancy.

### Healthy behaviors

Smoking and alcohol consumption are two recognized risk factors that can be modified through appropriate pregnancy education programs. Based on the well-established association between maternal smoking, alcohol intake, and poor birth outcomes (83–85), behavioral interventions are mostly conducted among women. Interestingly, among married Chinese couples, nearly one-third of fathers and only 3% of mothers consumed alcohol before pregnancy (29, 86). Further evidence from the database of the National Free Preconception Health Examination Project in China revealed that paternal smoking and alcohol consumption during the preconception period may increase the risk of birth defects, including congenital heart disease, limb abnormalities, and neural tube defects (85, 87); this suggests that parental smoking cessation and sensible alcohol consumption prior to conception should be emphasized.

## Discussion

The Chinese government has made substantial investments in preconception care services, which have benefited more than 73 million couples. High-quality clinical studies, such as the CARNATION study, indicate the clinically important effect of preconception-to-pregnancy care. By taking advantage of the large database of the National Free Preconception Health Examination Project, abundant original and compelling evidence has been published. For example, representative studies link preconception paternal smoking and alcohol intake to birth defects (87, 88) and reveal surprising protective effects of preconception folic acid supplementation to reduce spontaneous abortion (57); these great successes have gained wide attention. They further demonstrated the necessity of a nationwide-free preconception care strategy in China.

However, preconception care in China has some limitations, and the following aspects may indicate directions for future optimization. First, the integration of preconception and antenatal care must be strengthened. The continuum of care may have the potential to promote maternal health (89), as conventional prenatal care excludes the critical period of fetal organogenesis to provide primary interventions (before the 12th gestational week). In addition, the appropriate arrangement of preconception health examinations and premarital medical examinations should be considered, to thereby improve cost-effectiveness. Second, the implementation of preconception care in urban areas should be improved. Nearly 42.2% of couples in Shanghai, one of the most prosperous cities in China, report that they have attended preconception care, and the rate of intended pregnancy is still relatively low (90, 91). Raising awareness of planned pregnancy and preconception health will enable the implementation of preconception care services. At the same time, actions to address the uneven distribution of wider determinants, such as housing, education, and family income, and the provision of additional support for families with socioeconomic deprivation (91, 92) are required for a universal preconception care strategy in China. Third, associated local guidelines in terms of common infections, immunization, and pre-existing medical conditions should be updated, based on more population-based evidence. More focus is needed for proper inter-pregnancy interval and re-pregnancy management in “the post-cesarean section era” (93), which affects pregnancy outcomes and is an important issue for discussion in Western countries. In addition, genetic interventions through new technologies in pre-pregnancy screening, an important focus point in current research (94), may be specifically recommended for high-risk couples. Finally, with the rapid development of fifth-generation mobile communication technology and artificial intelligence, personalized preconception care services will be the future direction. Mass data concerning preconception health examination, prenatal care, and puerperal and newborn outcomes will be stored in a cloud server and automatically matched by a unique ID to establish a personal standardized profile of reproductive health and family planning in the future. Big data analysis will enable researchers and policymakers to adjust their local strategies based on evidence.

## Summary

Preconception care in China is a robust public health program that runs a universal implementation model. Furthermore, its valuable database has provided epidemiological evidence for the necessity and effectiveness of preconception care, to some extent. The successful experience of maternal and children's health service initiatives in China is available in this review to inform policymakers in other countries around the world.

## Author contributions

JX, XL, and QZ contributed to the study conception and design. JX searched the databases, collected the evidence, and wrote the first draft of the manuscript. XL and QZ revised the manuscript. All authors contributed to the manuscript 380 revisions, read, and approved the submitted version.

## Funding

This work was supported by the National Key Research and Development Program (Nos. 2021YFC2701600 and 2021YFC2701601), Shanghai Key Program of Clinical Science and Technology Innovation (Nos. 17411950500, 17411950501, and 18511105602), Clinical Research Plan of SHDC (Nos. SHDC2020CR1047B and SHDC2020CR6021), the Science Foundation of Shanghai (No. 21ZR1410600), Shanghai Excellent Young Scholar Plan of Public Health (2020-2022, GWV-10.2-YQ13), Elite Young Scholar 2025 of Fudan University (2020-2023), the National Science Foundation of China (81741047), and Shanghai Medical Center of Key Programs for Female Reproductive Diseases (No. 2017ZZ01016).

## Conflict of interest

The authors declare that the research was conducted in the absence of any commercial or financial relationships that could be construed as a potential conflict of interest.

## Publisher's note

All claims expressed in this article are solely those of the authors and do not necessarily represent those of their affiliated organizations, or those of the publisher, the editors and the reviewers. Any product that may be evaluated in this article, or claim that may be made by its manufacturer, is not guaranteed or endorsed by the publisher.
